# Why men who have sex with men in New Zealand intend to donate or not donate blood

**DOI:** 10.1111/vox.70043

**Published:** 2025-05-12

**Authors:** Carl Webber, Koson Tony Sriamporn, Sarah L. Morley, Stephen Ritchie, Brooke M. Hollingshead, Susan McAllister, Patricia Priest, Mark Fisher, Peter Saxton

**Affiliations:** ^1^ School of Population Health, University of Auckland Auckland New Zealand; ^2^ New Zealand Blood Service Auckland New Zealand; ^3^ School of Medicine, University of Auckland Auckland New Zealand; ^4^ Burnett Foundation Aotearoa Auckland New Zealand; ^5^ AIDS Epidemiology Group, Department of Preventive and Social Medicine University of Otago Dunedin New Zealand; ^6^ Body Positive Inc. Auckland New Zealand

**Keywords:** blood donation policy, intention, men who have sex with men, New Zealand, thematic qualitative analysis

## Abstract

**Background and Objectives:**

As blood donor deferral policies in many countries transition from blanket time‐based approaches towards individualized risk‐based approaches, blood services need to understand whether and why men who have sex with men (MSM) intend to donate blood. Such knowledge can help blood services develop communication strategies with a population they have historically excluded. We examined why MSM in New Zealand (NZ) intended or not to donate blood.

**Materials and Methods:**

In a large and diverse NZ online human immunodeficiency virus behavioural surveillance survey (*n* = 3838), we asked participants: ‘In your own words, please tell us why you intend to or don't intend to donate blood in the future?’ We interpreted 2363 responses by MSM using reflexive thematic analysis.

**Results:**

We constructed four themes to explain why MSM intend to donate blood: (1) helping out by donating blood, (2) donor ineligibility and hesitancy, (3) considerations of individual and collective risk and (4) discrimination, exclusion, frustration and resentment. Our study showed that whether or not MSM in NZ assessed blanket deferral policies as discriminatory, many MSM in NZ were altruistically and civically motivated to donate blood. Some MSM were reluctant to donate because they were resentful or mistrustful of the New Zealand Blood Service (NZBS) or believed that donating blood was inconsistent with their indigenous values.

**Conclusion:**

Further research is required to enhance communication, improve MSM's understanding of the donation process and residual risks and accommodate the cultural values of prospective indigenous donors.

**Highlights**

- Whether or not they assessed blanket deferral policies as discriminatory, many men who have sex with men (MSM) in New Zealand (NZ) were altruistically and civically motivated to donate blood.
- Some MSM in NZ were reluctant to donate because they were resentful or mistrustful of the New Zealand Blood Service or believed that donating blood was inconsistent with their indigenous cultural values.
- Research is required to enhance communication, improve MSM's understanding of the donation process and associated risks and accommodate the cultural values of prospective indigenous donors.

## INTRODUCTION

Blood services face the challenge of implementing evidence‐based changes to increase the blood supply while maintaining safety standards and stakeholders' trust [1]. Increasing the donor pool of men who have sex with men (MSM) could help reduce supply shortages, with an estimated 77%–82% of MSM wanting and intending to donate blood if eligible [1, 2]. Consequently, as human immunodeficiency virus (HIV)‐testing technologies have improved, blood service providers have incrementally reduced blanket deferral periods for MSM and are transitioning towards individualized risk assessments [3, 4, 5, 6].

Understanding the reasons behind prospective blood donors' intentions can inform recruitment and retention strategies [7]. Many donors identify altruistic motivations and a desire to engage civically as underpinning their intentions to donate [8, 9, 10, 11, 12, 13, 14, 15]. These have resulted in blood donation recruitment messages that implore potential donors to give the gift of life and save lives, and highlight donor shortages when advertising blood donor drives [16, 17, 18, 19].

However, recent studies suggest that MSM may engage differently with blood services. For example, some MSM donors knowingly did not comply with deferral policies, citing perceived unfairness towards MSM and the broader gay community [1, 20, 21]. MSM's understanding of blood donation is complicated by their engagement with community discussions about blanket deferral policies. Quantitative studies from Canada, Australia and New Zealand (NZ) suggest that MSM view these policies as unfair and homophobic, given the increasing adoption of biomedical HIV‐risk‐reduction approaches [1, 20, 22].

Recent qualitative studies have aimed to explain how MSM's understanding of blood donation might translate into willingness to donate [1, 19, 22]. Like non‐MSM donors, MSM understand donating blood as a charitable act or an opportunity to engage civically [1, 11, 12, 17, 19, 23]. Such studies typically explain MSM's understanding of blood donation and attitudes towards donation policy with reference to disenfranchisement [17, 19, 24, 25]. While they have described what blood donation means to MSM and why MSM have a ‘general positive affinity’ towards blood donation [19], they have not examined how MSM's understanding of blood donation informs their intention to donate.

Like its international counterparts, the New Zealand Blood Service (NZBS) must understand why MSM intend to donate to develop communication strategies and reinforce MSM's intentions to donate as blood services transition from blanket time‐based policies towards more inclusive, individualized risk policies [26]. Our study examines why MSM intend to donate or not in the future.

## MATERIALS AND METHODS

The Sex and Prevention of Transmission Study (SPOTS) is NZ's online bio‐behavioural surveillance programme focused on HIV prevention. The methods are reported elsewhere [2, 20, 27]. In brief, participation was voluntary, anonymous and self‐completed. Participants were eligible for the study if they were aged 16 or over, living in NZ, a man (cis or trans) who had ever had sex with a man or identified as gay, bisexual, takatāpui (Māori LGBTQIA+), queer or pansexual, or were transgender or non‐binary and had sex with men in the previous 5 years. Consent was obtained before completing the online survey. Ethics approval was received from the NZ Health and Disability Ethics Committee (HDEC 2021 EXP 11450). The survey was conducted from April to August 2022, following the relaxation of the MSM deferral in December 2020 from 12 to 3 months and when blood donation was politically salient for MSM.

Participants were asked about blood donation histories, awareness of donation deferral rules and their interest and attitudes towards donation. Participants were informed about the current policy without providing any rationale [2, 27]. Participants were also asked whether they would donate blood should they become eligible. The quantitative findings are summarized elsewhere [2, 28]. Reasons for why participants intended to donate or not in the future were collected through a single, open‐ended item, where individuals responded to the question: ‘In your own words, please tell us why you intend to or don't intend to donate blood in the future?’ Participants were not required to answer this question, and the textbox had no character limit.

We conducted a scoping literature review on the motivations for MSM blood donation. We analysed the responses using reflexive thematic analysis (RTA) [29]. RTA is a flexible method that allows inductive analysis of large datasets and coheres with our understanding of intentions as socially constructed [30, 31]. All written responses were de‐identified and provided for qualitative analysis with information about each participant's age cohort (16–19, then deciles to age 69, then 70–74) and ethnicity (New Zealand European [NZE], indigenous Māori, Pacific ethnicities, Asian, Middle Eastern, Latin American or African [MELAA] and ‘Other’) [2]. The lead author (C.W.) inductively generated 45 initial codes using QSR Nvivo 14. Team members cross‐checked the codes. C.W. constructed themes, which were reviewed by team members (outside Nvivo using mind‐mapping software X‐Mind PRO) to ensure they reflected participant perspectives.

## RESULTS

Overall, 3838 participants completed the online survey. Of these, 2363 were MSM and responded to the open‐ended item about blood donation intentions.

We constructed four themes to explain why MSM intend to donate blood or not: (1) helping out by donating blood, (2) donor ineligibility and hesitancy, (3) considerations of individual and collective risk and (4) discrimination, exclusion, frustration and resentment.

### Helping out by donating blood

In our study, MSM who intended to donate blood were commonly altruistically or civically motivated. Participant reasons for donating were often complex, drawing on multiple aspects of the theme. Many MSM expressed a desire to ‘help save lives’ or ‘help others that may need blood’.

For some participants, considerations of need informed their charitable intention to donate blood. Many participants expressed concern about the persistent demand for more supply. As one explained, ‘There is often a struggle with having enough supply in the blood banks’ (20–29, NZE). Echoing this sentiment, another participant emphasized that blood was ‘desperately needed’ (20–29, NZE). For some, this shortage was compounded by the limited number of donors, with one participant pointing out, ‘There is a shortage of blood donors’ (20–29, NZE). Others reflected on the personal significance of their blood types, with someone acknowledging, ‘Our health system needs all the help it can get, and I have a rare blood type’ (30–39, NZE).

Many participants intended to donate as an act of upstream or downstream reciprocity. One participant said, ‘I would hope that if I can help someone, they would do the same for me’ (40–49, Māori). Another reflected, ‘I have whānau (family) who have survived due to being donated blood. I would like to give back’ (30–39, NZE).

The intentions of some MSM to donate were rooted in civic responsibility. Some felt obliged to donate blood. One participant said, ‘an active participant in our society in Aotearoa … It's like paying taxes—everyone should be doing it’ (50–59, NZE). Several expressed their motivation to donate using rights‐based language. ‘As a civic duty, I want to donate blood to feel as though I am an active participant in our society in Aotearoa (NZ)’. Others framed their motivation to donate in less obligatory terms, construing it as a community or social contribution. For example, one participant stated, ‘I want to donate blood for the good of others and to contribute as a citizen of Aotearoa New Zealand’ (NZE, 60–69). One Māori man claimed, ‘I'm a socially minded person and see it as a form of community service’ (20–29, Māori). Another said, ‘I want to awhi [support] my hapori [community]’ (20–29, Māori).

### Prospective donor ineligibility and hesitancy

Participants commonly cited their reasons for intending to not donate as ineligibility. Old age and a history of travel to the United Kingdom during the 1980s (with associated risk of Creutzfeldt–Jakob disease exposure) were correctly cited as exclusionary criteria (60–69, NZE; 30–39, NZE). Participants living with HIV (PLHIV) recognized that the risk of transmitting HIV via blood transfusion meant they were ineligible to donate. One admitted, ‘Being undetectable still carries a margin of risk’ (40–49, Asian). Another lamented, ‘HIV is a horrible disease. I don't intend to donate blood with HIV’ (40–49, NZE). Another participant drew from the Māori concept of ‘tapu’ (‘sacred’ or ‘spiritually restricted’ [33]), reasoning that the virus needed to be ‘contained’ within him to protect his community: ‘I'm HIV positive and undetectable, I feel best to not donate my blood, it's tapu to me’ (40–49, Māori).

Despite being ineligible, many PLHIV in our study were keen to explore alternative ways to donate blood products without compromising the safety of the blood supply. One participant suggested, ‘If my +U blood was being used for the treatment of another +U person, I would donate happily’ (50–59, NZE).

Other participants did not intend to donate if it required abstaining from anal intercourse or foregoing pre‐exposure prophylaxis (PrEP). While expressing their determination to adhere to the current MSM‐deferral policy, some noted that abstaining from anal intercourse for 3 months was unfeasible. ‘I would love to give blood but abstaining from anal sex for three months is unrealistic for me, so I can't’, said one man (20–29, NZE). Another dismissed the notion of giving up PrEP: ‘I can't donate any more as I'm on PrEP daily and am not giving that up as I wish to keep myself safe from HIV infection’ (50–59, NZE).

Some MSM expressed apprehension about the process of donating blood, which grounded their intention not to donate. Many expressed a ‘discomfort with needles’ (20–29, NZE). A teenage participant explained, ‘[I'm] nervous about getting blood taken’ (16–19, NZE). Other MSM expressed concerns about disclosing their sexual history to NZBS staff. One said, ‘I want to donate blood but am uncomfortable always being open about my sexual history’ (20–29, NZE).

Some Māori participants appealed to Te Ao Māori (the Māori worldview) to explain why they intended not to donate blood. One participant told us that because blood was ‘mauri’ (the essential quality or vitality of being), he would not share it with those with whom he did not share a common ancestry, stating, ‘Because in Te Ao Māori everything has a mauri [essential quality and vitality of being], meaning I wouldn't want to pass some of my mauri into another human that isn't a direct whakapapa [genealogical link] to me’ (20–29, Māori).

A few participants expressed apathy about donating blood or did not consider or personally prioritize it. One participant explained, ‘[Blood donation is] not a priority for me at the moment’ (30–39, NZE), and another, ‘it's something that does not really enter my mind’ (60–69, NZE).

### Considerations of individual and collective risk

Several participants argued that MSM as a population represented equivalent or less risk than their heterosexual counterparts. One noted, ‘Straight people have a lot of random sex, too, but can still donate’ (40–49, NZE). Another pointed to the increased likelihood that MSM who identify as heterosexual are less likely to disclose their sexual histories accurately, claiming that it was ‘incredibly hypocritical given the amount of married men in “straight” relationships who engage in sex with men and aren't exactly likely to declare that on a form’ (30–39, NZE).

In contrast, others focused on their individual risk to the blood supply, arguing that as individuals, they engaged in low‐risk sexual behaviour and adhered to regular testing. ‘Regular screening practicing safe sex being confident your MSM partner is transparent and has the eligible proof of his recent screening test’ ([age‐unknown], NZE). Another stated, ‘As a guy in a long‐term monogamous relationship, I am extremely low risk, so should be permitted’ (40–49, NZE).

Importantly, several participants also assumed that testing all blood for sexually transmitted infections (STIs) was sufficient to protect the blood supply from HIV. According to one man, ‘While quite sexually active, I assume that blood is tested for disease before being given …’ (20–29, NZE). Another told us, ‘I'd donate, but I feel like I'm being judged, and I'm sure testing would show up anything before blood was distributed’ ([age‐unknown], NZE).

### Discrimination, exclusion, frustration and resentment

Many participants perceived blanket deferral policies to be discriminatory because they determined donor risk based on their being MSM rather than assessing individual behaviours. One participant suggested that since ‘blood gets tested … the rules should be the same for everyone’ (40–49, Asian). Another protested, ‘The current policy is completely out of line’ (20–29, NZE). One explicitly labelled his resentment, ‘I feel resentment for becoming ineligible despite practicing safe sex’ (20–29, Māori). Another said, ‘To be honest, I feel a bit sour about the whole not being able to donate thing’ (20–29, NZE). Others were franker about their feelings. ‘Suddenly, our dirty blood is good enough for you straight people to use. No thanks!’ said one man (30–39, Māori). ‘They haven't exactly rolled out the red carpet’ objected another (30–39, NZE).

The current MSM deferral policy left some participants feeling socially excluded: ‘I want to be able to help others and donate blood like any other Nzer and not be told I can't do something 'cause I'm gay’ (20–29, Māori), and ‘I feel like I haven't been able to contribute all my life because of being gay—it makes me feel less of a citizen by not giving back’ (50–59, NZE). Others expressed a sense of being stigmatized. One man said, ‘I think that it is an easy and important public service that actually saves lives. Not being able to makes me feel dirty and second class’ (30–39, NZE).

Understanding the current blanket deferral policy as discriminatory or exclusionary did not determine whether participants intended to donate. For some, their sense of being socially excluded provided a reason to donate—to be socially included. One participant stated, ‘I want to be like everyone else and get the chance to donate blood’ (20–29, Māori). Another wanted to donate to improve his social standing: ‘I like the idea of contributing to society. It makes me feel like I am as good as straight people. This is why I intend to donate’ (50–59, NZE). Others expressed frustration at being ineligible under current policies but still wanted to donate: ‘I assume they don't want my blood. Otherwise, I would donate today. The current exclusion is not based on science—and we need blood donations’ (50–59, NZE). Other participants looked forward to fairer rules that allowed them to donate, with one participant stating, ‘One day risk may be calculated based not on sexuality but actual risk activities, two monogamous non‐drug‐taking negative men can't spread HIV’ (40–49, NZE).

In contrast, other participants' negative evaluations of blanket deferral policies left them reluctant to donate: ‘Because of being unable to for so long it makes me feel bitter and that I won't out of spite if I'm ever able to’ (20–29, NZE). Another said, ‘For so long they have not wanted my perfectly fine blood, so a change in policy would leave me hesitant. Why do they now want my blood when it's been the same all along? Kind of a “stuff you” mentality’ (40–49, NZE). One man expressed that the blanket deferral caused him ‘deep pain and anger’ (20–29, Māori). Another stated, ‘I feel like I won't ever be able to trust them’ (20–29, NZE). Others remained ambivalent about their intention to donate but recognized the need to acknowledge historical harm: ‘There's a bit of historic prejudice that I'd want to see acknowledged’ (40–49, Māori).

## DISCUSSION

The MSM who participated in a NZ HIV behavioural surveillance survey reported a variety of reasons why they intended or did not intend to donate blood in the future, should they become eligible. We identified four themes: helping out by donating blood; donor ineligibility and hesitancy; considerations of individual and collective risk; and discrimination, exclusion, frustration and resentment. Participants' donation rationale generally did not differ by age or ethnicity. Most participants reported wanting to contribute, but many expressed frustration with current policies and self‐assessed their risk as low. Other participants had no intention of donating because of their resentment or mistrust of the NZBS or because they believed donating blood contradicted their indigenous cultural beliefs.

Our findings are consistent with international research, which demonstrates that, like all blood donors, MSM understand donating blood as an act of charity or civic engagement [1, 8, 11, 12, 13, 14, 17, 19]. Our research shows that MSM in NZ were charitably and civically motivated to ‘help out by donating blood’. Our research also shows how MSM in NZ perceived their ‘individual and collective risk’ informed their understanding that the current blanket deferral policy was ‘unfair and discriminatory’. This finding is consistent with international findings, which suggest that blood donation for MSM can be understood as the interface between sexual and biological citizenship [17, 19]. According to these accounts, MSM actively engage with government agencies to advocate for their full participation as citizens by donating blood, which is seen as a form of ‘sexual citizenship’ [34, 35]. MSM also engage in ‘biological citizenship’ projects [24], collaborating with blood services and community stakeholders to determine acceptable risks associated with blood safety [17, 19, 25, 36]. Grace et al. appeal to sexual and biological citizenships to explain why MSM have a positive affinity to blood donation [19]. Our study adds to current international research by examining why MSM intend to donate blood or not.

Our findings have several implications for blood donation policy. First, many participants expressed frustration and resentment towards NZBS, suggesting that reparation between NZBS and MSM is important. However, the appropriate actions required vary depending on the context. Consequently, blood services have adopted different approaches to reconciliation. For instance, while the United States and the United Kingdom transitioned smoothly from blanket deferrals to individualized risk assessments, the strong discontent expressed by LGBTQIA+ communities resulted in the Canadian Blood Services offering a formal apology ‘as a necessary step in our ongoing journey to build trust and repair relationships with individuals and communities impacted by the former donor eligibility policy’ [37]. To understand what response MSM in NZ believe is appropriate requires further qualitative research. In the meantime, the NZBS must continue its public engagement with MSM, including targeted communication of eligibility criteria and relevant risk considerations.

Second, some of our participants' claims of unfair treatment were based on misunderstandings of the risks associated with blood safety. For instance, some participants were unaware that blood screening does not eliminate the risk of pathogens being undetected due to the window period or that MSM have a relatively high probability of undiagnosed HIV compared to other populations in NZ (even though this is very low in absolute terms). Some PLHIV were unsure whether having an undetectable viral load meant that they were safe to donate or not. Identifying the reasons behind misunderstandings can inform further communications aimed at addressing the concerns of hesitant donors and promoting adherence [28]. This may provide an opportunity for blood services to partner with HIV prevention and rainbow community organizations to ensure consistent messaging.

Third, our study showed that, like many donors, MSM donors' apprehension about the blood donation process was a barrier [12, 14]. Also, some MSM were anxious about the requirement to share their personal sexual histories. Like their Canadian counterparts, MSM in NZ might fear the potential ‘embarrassment’ of disclosing their sexual histories to blood service staff [26], unnecessarily violating their privacy, or that such disclosure equates to ‘coming out’ as gay, which they are reluctant to do in the context of blood donation. The NZBS must work with MSM prospective donors to understand how to mitigate their concerns and potentially increase the number of blood donors.

Fourth, our results highlighted the importance of cultural values in shaping how MSM engage with blood services. Some indigenous Māori participants' intention not to donate blood was informed by Te Ao Māori (Māori cultural worldview). As blood contains DNA—the physical embodiment of whakapapa—for some takatāpui in our study, blood was considered taonga, warranting its protection [33]. Consequently, Māori participants were reluctant to donate to a national blood supply. Blood services must accommodate the sensitivities of indigenous populations [28]. For the NZBS, this means more research is required to determine how best to accommodate the cultural values and lived experiences of prospective takatāpui donors to better understand how to increase the takatāpui donor pool. This would also offer more takatāpui access to the personal benefits of being a blood donor.

This analysis has several strengths. The SPOTS study provided the largest and most diverse sample of MSM in NZ, a population deeply affected by blood donation policies and challenging to sample using conventional methods. The anonymous and online completion mode may have also contributed to the openness of our participants' responses compared to interviewer‐administered questionnaires or evaluations conducted by blood service staff. Our research team comprised academics from diverse backgrounds in epidemiology, virology, psychology, public health and philosophy. It included members from HIV prevention and gay community organizations, as well as the NZBS. Several team members identified as gay men, while one identified as Māori; such diversity promoted reflexivity of positionality [29, 32]. We drew on our team's various ages, ethnicities, academic backgrounds and lived experiences to inform our analysis and interpretation. For instance, our different disciplinary backgrounds supported our understanding of the different participants' perceptions of the risks associated with blood donation. There were also some limitations. The research question was posed in a quantitative survey; thus, participant responses were less comprehensive than in‐person interviews or focus groups.

In conclusion, our study showed that whether or not MSM in NZ assessed blanket deferral policies as discriminatory, many MSM in NZ were altruistically and civically motivated to donate blood, but not all. Some MSM were reluctant to donate because they were resentful or mistrustful of the NZBS or believed that donating blood was inconsistent with their indigenous cultural beliefs. Further research is required to enhance communication, improve MSM's understanding of the donation process and associated risks, accommodate the cultural values of prospective indigenous donors and understand whether or what reparation might be appropriate in NZ.

## CONFLICT OF INTEREST STATEMENT

The authors declare no conflicts of interest.

## Data Availability

Research data are not shared.
